# Biological function of astaxanthin and its application in aquatic animal feeding^☆^

**DOI:** 10.1016/j.fochx.2025.103319

**Published:** 2025-11-26

**Authors:** Ying Zhu, Xiaoqi Li, Shengjun Wu

**Affiliations:** a Jiangsu Key Laboratory of Marine Bioresources and Environment/Jiangsu Key Laboratory of Marine Biotechnology, Jiangsu Ocean University, Haizhou 222005, China.; b Co-Innovation Center of Jiangsu Marine Bio-industry Technology, Haizhou 222005, China

**Keywords:** Astaxanthin, Aquaculture, Carotenoids

## Abstract

Astaxanthin, a carotenoid, is present in the shells of marine organisms and the tissues of fish, contributing to the characteristic reddish color of fresh fish due to its accumulation. It is primarily used as a natural pigment in aquatic organisms, with higher astaxanthin levels indicating superior quality in aquatic products. In aquaculture, astaxanthin enhances pigmentation, promotes growth and proliferation, improves immune function, and exhibits antioxidant properties. Its inclusion in feed can strengthen immunity and enhance the nutritional and commercial value of aquatic species. In medicine, astaxanthin is recognized for its antioxidant and immune-modulating properties. Additionally, it has been incorporated into cosmetics to mitigate allergic reactions induced by ultraviolet radiation, reduce radiation-induced damage, and provide antioxidative benefits.

## Highlights

• Astaxanthin has varying biological activities.
• Astaxanthin can improve the growth performance of aquatic animal.
• Astaxanthin can improve the immunity of aquatic animal.

## 1 Introduction

**Fig. 1:**
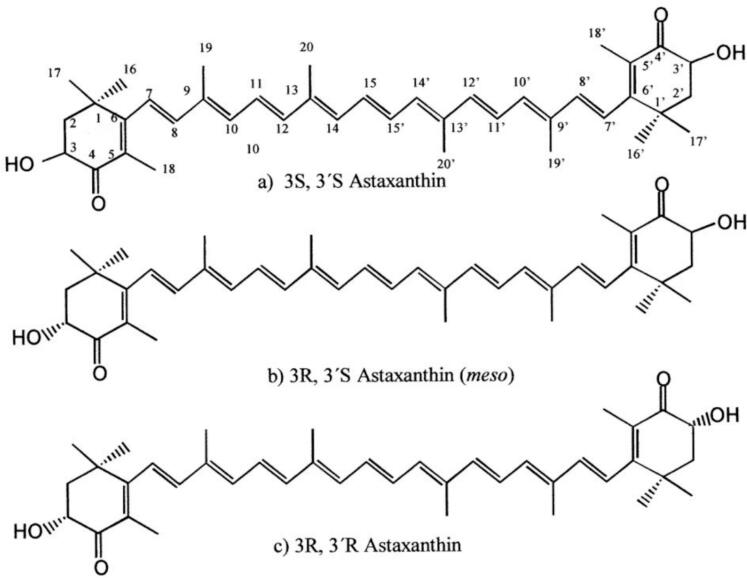
The chemical structure of astaxanthin.

Astaxanthin, a ketocarotenoid synthesized by microalgae such as *Haematococcus pluvialis* and stored in marine organisms, plays a critical role in aquaculture due to its functions as a natural pigment and antioxidant (Ambati, Phang, Ravi, & Aswathanarayana, 2014; Debnath et al., 2024; Oninku, Lomas, Burr, & Aryee, 2025). Its unique hydroxyl and ketone groups facilitate exceptional free radical scavenging, exceeding β-carotene and lutein in mitigating oxidative stress (Fig. 1) (Li, Li, Xu, Yang, & Li, 2024; Xu et al., 2024). In aquaculture, dietary supplementation (60–200 mg/kg) improves pigmentation in ornamental fish and shrimp, thereby enhancing market value, whereas also improving survival rates and immunological responses in species such as rainbow trout (Chen, Wang, Feng, & Xuan, 2023; Zhao et al., 2019). Recent studies (Khan, 2025; Li et al., 2025; Li, Yu, Wei, Liufu, & Su, 2025) highlight that astaxanthin plays a pivotal role in reducing oxidative stress and improving the immune system of aquaculture species. Additionally, Li, Yu, et al. (2025) demonstrated that astaxanthin supplementation significantly enhances growth performance and feed utilization in various aquaculture animals through a meta-analysis. Despite its benefits, 95 % of commercial astaxanthin is synthetically produced, raising concerns regarding isomer purity (Khadilkar, Karande, Prakash, & Pandit, 2023). Advances in bioreactor cultivation of *H. pluvialis* aim to enhance natural yields; however, challenges persist in standardizing dosages and addressing bioavailability variations among species (Kohandel, Farkhondeh, Aschner, Pourbagher-Shahri, & Samarghandian, 2022; Wang et al., 2024). Addressing these deficiencies is crucial for meeting the 24 % annual demand increase and fostering sustainable aquaculture (Mutale-Joan & El Arroussi, 2023). Aquaculture is growing rapidly, and antibiotic use is being restricted, thus sustainable, natural alternatives that improve animal health and product quality are needed. Therefore, this review consolidates contemporary astaxanthin research on biological functions, extraction methodologies, and aquaculture feeding applications. It explores astaxanthin's physicochemical properties, classic and innovative extraction methods, and pigmentation, antioxidant defense, growth promotion, and immunological enhancement in aquatic species. The review also examines present issues and future perspectives for industrial astaxanthin use, providing a comprehensive resource for academics and practitioners.

In recent years, global demand for natural astaxanthin in aquaculture has surged, exhibiting an annual growth rate of approximately 24 %, propelled by the rising necessity for functional feed additives that enhance immunity and survival in aquatic organisms (Market Research Future, 2023). This trend is further bolstered by regulatory limitations on antibiotic application in aquaculture. The European Union (2019) explicitly forbids the prophylactic use of antibiotics in feed, catalyzing a worldwide transition to natural, residue-free substitutes like astaxanthin. Nonetheless, the elevated expense and constrained production capacity of natural sources (e.g., *Haematococcus pluvialis*) continue to pose significant barriers to wider use. Confronting these problems via biotechnological advancements and economical production techniques is crucial for fostering the sustained advancement of the aquaculture sector.

## 2 Physical and chemical properties of astaxanthin

Astaxanthin is a carotenoid characterized by the presence of a ketone group. Its chemical designation is 3,3′-dihydroxy-4,4′-diketone group-β,β’-carotenoid, with the molecular formula C_40_H_52_O_4_. Astaxanthin manifests as a solid red powder, exhibiting solubility in fats whereas being insoluble in water, yet soluble in organic solvents (Yuan, Peng, Yin, & Wang, 2011). Astaxanthin is predominantly found in the feathers of various organisms, including avian species and poultry, where it serves primarily as a pigment. Compared to other carotenoids, astaxanthin possesses a distinctive structure, enhanced esterification capacity, and notable antioxidant properties (Debnath et al., 2024). These attributes are largely attributable to the hydroxyl and ketone groups within its molecular architecture. The pronounced antioxidant capacity of astaxanthin is a result of its function as a reducing agent and its robust electron-donating capability, which allows it to neutralize free radicals, including hydroxyl radicals, anions, and hydrogen peroxide, transforming them into more stable entities (Xu et al., 2024). Astaxanthin primarily interacts with one or two proteins or fatty acids, such as those found in crustacean shells, and has been shown to enhance the stability of salmon muscle (Saha, Ross, Olsen, & Lall, 2006). Therefore, astaxanthin demonstrates considerable potential for application in the formulation of nutritional diets and the promotion of animal health.

**Fig. 2:**
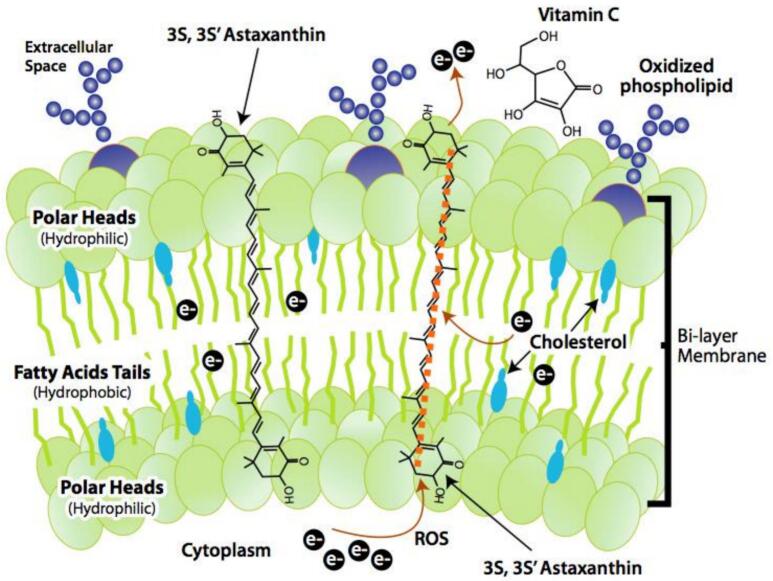
Biological activities of astaxanthin. Astaxanthin exerts potent antioxidant effects by quenching free radicals, enhances immunity, reduces inflammation, protects mitochondria, and supports neurovisual health through multiple molecular pathways.

Astaxanthin is also acclaimed for its antioxidant properties. Li et al. (2024) conducted investigations into the effects of astaxanthin on reactive oxygen species, antioxidant capacity, and gene expression related to molting in the larvae of *Macrobrachium rosenbergii*, a prominent freshwater shrimp species. These findings confirmed that astaxanthin effectively mitigates sulfides, reduces lipid levels, and inhibits lipid peroxidation driven by free radicals. A recent study by Li, Chen, et al. (2025) and Li, Yu, et al. (2025) further substantiated that dietary astaxanthin significantly improves growth, coloration, immunity, and antioxidant capacity in *Macrobrachium rosenbergii*. Furthermore, astaxanthin demonstrates efficacy in tumor inhibition, immune system enhancement, and improvement of physical fitness in humans. It exhibits therapeutic effects on allergic reactions and symptoms induced by ultraviolet exposure, as well as significant preventative effects against diabetes-related ocular complications, including diabetic retinopathy and macular edema, which are characterized by vascular damage and oxidative stress within retinal tissues (Fig. 2). Given these advantageous effects, astaxanthin holds substantial promise for applications across food, medicine, health, beauty, and aquaculture sectors (Li, Xie, Zeng, & Khan, 2023).

## 3 Extraction of astaxanthin

### 3.1 Distribution forms of astaxanthin

Astaxanthin is present in various organisms and tissues, contributing to their stability. It exists in two primary forms: esterified and free. Esterified astaxanthin, which remains unoxidized, is predominantly found in shelled organisms such as fish, shrimp, and crabs. Conversely, free astaxanthin is primarily located in internal organs, such as the liver and kidneys. Astaxanthin exists in two primary forms: esterified and free. Esterified astaxanthin is predominantly found in shelled organisms such as fish, shrimp, and crabs, while free astaxanthin is primarily located in internal organs (Higuera-Ciapara, Félix-Valenzuela, & Goycoolea, 2006).

### 3.2 Advanced extraction methods for astaxanthin

Astaxanthin can be extracted by several innovative techniques aimed at enhancing efficiency and production from natural sources, including *Haematococcus pluvialis* microalgae (Ambati et al., 2014; Debnath et al., 2024; Oninku et al., 2025). The principal methods for astaxanthin extraction encompass solvent extraction, supercritical CO_2_ extraction, and enzymatic-assisted extraction. Solvent extraction is a prevalent technique that provides reasonably high yields, typically ranging from 3 to 5 % of the dry weight of *H. pluvialis* (Ambati et al., 2014). This approach, although effective, necessitates meticulous solvent selection to prevent the breakdown of astaxanthin. Supercritical CO_2_ extraction, an eco-friendly and solvent-free method, delivers high-purity astaxanthin at rates of 7–8 % from the dry weight of *H. pluvialis* (Ambati et al., 2014; Kim, Lee, Narasimhan, Kim, & Oh, 2022). Nonetheless, its elevated operational expenses and energy usage constrain its extensive implementation. Enzymatic-assisted extraction, which increases yield by degrading the cell walls of microalgae, can enhance extraction efficiency by 20–30 %, attaining yields of up to 10 % under optimum circumstances (Kim et al., 2022). Moreover, genetic engineering and gene-editing methodologies are utilized to enhance the production of astaxanthin in *H. pluvialis*, augmenting the astaxanthin concentration without affecting the extraction process. These methods can achieve yields of 12–15 % in genetically engineered strains of *H. pluvialis*, albeit they prioritize production enhancement above extraction. These sophisticated procedures are essential for enhancing astaxanthin yield, and ongoing research is necessary to optimize these methods for more efficient large-scale manufacturing (Khadilkar et al., 2023; Oninku et al., 2025; Wang et al., 2024).

Recent innovations in astaxanthin extraction methods have led to the use of sustainable cultivation practices, such as recirculating aquaculture effluent (Timilsina et al., 2025), which could significantly lower the costs and environmental impact of astaxanthin production.

#### 3.2.1 Extraction of astaxanthin via chemical synthesis

The high demand for astaxanthin has motivated research efforts, which may be classified into two primary methodologies: chemical synthesis and biological extraction. The chemical extraction of astaxanthin includes two approaches: direct synthesis and indirect synthesis. Direct synthesis is typically used for carotenoid production, whereas indirect synthesis requires the oxidation of other carotenoids before synthesis. A notable drawback of the chemical synthesis method is its complexity, necessitating the initial extraction of carotenoids, resulting in astaxanthin existing in a comparatively less stable free state (Hamdi et al., 2022) (Table 1).

**Table 1:** Comparison of astaxanthin extraction method.

Method	Yield Improvement/Content	Advantages	Limitations	References
Solvent Extraction	3–5 % from dry weight of *Haematococcus pluvialis*	Simple and cost-effective	Requires careful solvent selection; lower purity	Ambati et al. (2014)
Supercritical CO_2_ Extraction	7–8 % from dry weight of *Haematococcus pluvialis*	High purity; solvent-free	High operational cost; energy consumption	Brivio, Schlosrich, Ahmad, Tolond, and Bugg (2009)
Enzymatic-Assisted Extraction	10 % under optimized conditions	Enhances extraction efficiency by breaking cell walls	High enzyme cost; suitable for small-scale	Brivio et al. (2009)
Genetic Engineering	12–15 % improvement in *Haematococcus pluvialis*	Increased astaxanthin content in modified strains	Not an extraction method; focuses on production	Ambati et al. (2014)
Improved Extraction with Dual Solvent	Up to 10 % with dual solvent extraction (Ethanol and acid treatment)	Improved efficiency and yield under optimized conditions	Requires multiple extraction steps; solvent residue	Shi et al. (2021)
Microbial Fermentation for Enhanced Yield	Up to 15 % improvement in natural astaxanthin content from microalgae	Efficient continuous production; cost-effective	Slow process; dependence on strain efficiency	Khadilkar et al. (2023)

#### 3.2.2 Astaxanthin obtaining via the biological extraction method

The biological extraction method for obtaining astaxanthin primarily relies on the utilization of aquatic product waste as a raw material. Current international practices for astaxanthin extraction use polymeric agents to obtain astaxanthin from waste sources such as shrimp and crabs. This method enhances the value of astaxanthin and effectively transforms waste into a valuable resource. The production value can reach up to 153 g/L (Santos et al., 2012), thereby alleviating the burden of waste treatment (Dutta, Kumar, & Banerjee, 2023). In recent years, silage has also been employed internationally to manage industrial and fishery waste. Originally used for feed preservation, silage undergoes fermentation and acid production from raw materials, effectively inhibiting the growth of harmful microorganisms (Ambati et al., 2014) (Table 1).

Research has demonstrated that by comparing various methods and adjusting solvent types and concentrations, the highest content of natural astaxanthin can be extracted from shrimp shells through techniques such as ethanol extraction, acid pretreatment, decalcification, and the implementation of dual extraction processes (Wang, 2005). Furthermore, single-factor experiments and orthogonal experiments have indicated that factors such as pH, temperature, and time significantly influence the efficiency of natural astaxanthin extraction at a solid–liquid ratio of 1:10, pH 3.0, and a temperature of 60 °C. The optimum extraction effect is achieved after 3.5 h (Shi et al., 2021) (Table 1).

Astaxanthin is sensitive to heat and light. Research demonstrates that degradation markedly intensifies at temperatures over 70 °C, especially with extended exposure. At 80 °C, astaxanthin degradation may surpass 30 % in just 2 h (Shi et al., 2021). Likewise, under intense lighting (e.g., 10,000 lx full-spectrum light), photooxidation markedly diminishes astaxanthin stability. Consequently, the temperature must be maintained below 50–60 °C, and extraction should occur in low-light, oxygen-restricted conditions to reduce oxidative deterioration during processing and storage.

The widely established ideal ethanol extraction conditions for shrimp shells (solid-liquid ratio 1:10, pH 3.0, 60 °C, 3.5 h) are influenced by variations in source material, which impact extraction performance. Crab shells possess elevated concentrations of calcium carbonate and chitin, necessitating preliminary decalcification (e.g., 0.5 mol/L HCl for 30 min) and a modified temperature of around 55 °C to prevent excessive protein denaturation. Conversely, shrimp shells possessing softer exoskeletons achieve enhanced astaxanthin yield at a somewhat elevated extraction temperature (60–65 °C) and reduced reaction time (Shi et al., 2021; Wang, 2005). These findings underscore the necessity for optimization tailored to unique source materials during biological extraction.

## 4 Application of astaxanthin in aquaculture

### 4.1 Promotion of coloring effects

The initial application of astaxanthin in aquaculture to enhance the coloration of aquatic organisms was reported in 1998, when Bjerkeng, Hatlen, and Wathne (2000) alternately fed fish with 1.4 mg/kg (feed 1) and 2.1 mg/kg (feed 2) astaxanthin to improve the nutritional value of salmon and rainbow trout. The astaxanthin content significantly increased. Additionally, due to the binding of astaxanthin to lipids, the concentration varies in different parts of the same aquatic organism and increases with elevated lipid content. The observed interspecies differences in astaxanthin utilization between rainbow trout and Atlantic salmon may be related to their differential capacities for intestinal absorption and metabolism. It has been hypothesized that variations in the expression levels of intestinal carotenoid transporters (e.g., SR-B1 and CD36), lipid assimilation efficiency, and esterification patterns of astaxanthin could contribute to these species-specific differences. Further genomic and transcriptomic studies are needed to validate these mechanisms.

Recent research (Sun et al., 2025) demonstrated that astaxanthin supplementation not only enhances coloration but also improves stress resilience and immune responses in largemouth bass. Similarly, Meng, Yang, Zhan, Numasawa, and Deng (2025) reported that dietary astaxanthin sources significantly impact the growth performance, antioxidant status, immune response, and intestinal morphology in rainbow trout (*Oncorhynchus mykiss*).

The coloring effect is widely utilized in ornamental fish, primarily through the incorporation of astaxanthin into their feed. This results in a more vibrant and glossy appearance whereas maintaining a natural coloration. The value of ornamental fish predominantly lies in their coloration and posture. In addition to factors such as light, water source, and temperature, feed plays a significant role in determining the coloration of a fish's body (Osawa, Nishi, Kuwahara, Haga, & Honda, 2024).

Mutale-Joan and El Arroussi (2023) conducted a study in which ornamental fish were administered a diet containing 100 mg/kg of natural astaxanthin (from *H. pluvialis*) for one week. The findings indicated a significant enhancement in the surface coloration of the fish (15 % higher ΔE value compared to synthetic astaxanthin at the same dose), with improved color vibrancy and transparency. This aligns with Chen et al. (2023), who reported superior carotenoid deposition in shrimp muscle using natural astaxanthin, likely due to its 3S,3′S configuration and higher bioavailability (Ambati et al., 2014). Moreover, Tran, Doan, Dang, Hua, and Pham (2025) demonstrated that dietary astaxanthin derived from shrimp shell waste enhances growth performance, coloration, and body composition in hatchery-produced false clownfish (*Amphiprion ocellaris*).

Most ornamental fish are bred artificially, leading to reduced levels of natural pigment components and less pronounced body coloration. In contrast, wild ornamental fish, which consume algae in their natural habitats, accumulate carotenoids containing astaxanthin in greater quantities, resulting in more vibrant and commercially valuable colors. Therefore, increasing the astaxanthin content represents an effective strategy for enhancing the vibrancy and transparency of ornamental fish (Chen, 2012). Zhao et al. (2019) provided goldfish with yeast enriched with high astaxanthin content and reported that the addition of 60 mg of astaxanthin per kilogram of feed produced the optimal coloring effect in goldfish. Spectroscopic analyses further confirmed that goldfish under these dietary conditions exhibited the most natural coloration.

Astaxanthin also significantly influences the coloration of shrimp. Given that the market value of shrimp is contingent upon their freshness and sensory quality, astaxanthin is widely regarded as the most effective coloring agent currently available. Pan and Chien (2003) conducted experiments in which Japanese shrimp were fed 100 mg/kg of astaxanthin, 100 mg/kg of keratin, or 100 mg/kg of β-carotene. The results demonstrated that shrimp fed astaxanthin exhibited increased carotenoid contents (Kim et al., 2022).

### 4.2 Antioxidant properties of astaxanthin

Research conducted by DiMascio, Kaiser, and Sies (1989) demonstrated that the singlet oxygen quenching activity of astaxanthin is 1.7–3.8 times greater than that of β-carotene and lutein. Astaxanthin exhibited the strongest antioxidant capacity, reducing lipid peroxidation by 42 % compared to the control group (Chen, Huang, et al., 2023). Pan and Chien (2003) further reported that astaxanthin at 100 mg/kg feed significantly protected phospholipid membranes in shrimp, inhibiting lipid peroxidation by 35 %. During transportation, Huang, Liu, Hu, Li, and Wang (2022) observed that rainbow trout fed 150 mg/kg astaxanthin retained 89 % muscle freshness after 48 h, compared to 62 % in the control group, due to reduced lipid oxidation. The quality of aquatic products deteriorates during transportation, with bacterial growth primarily attributed to lipid oxidation. By inhibiting lipid oxidation, astaxanthin can enhance the freshness of aquatic products. Therefore, the application of astaxanthin in the refrigeration preservation of raw meat can be expanded (Table 2).

**Table 2:** Antioxidant effects of astaxanthin on aquatic animal.

Subject	Dosage (mg/kg)	Key Findings	Source of Astaxanthin	References
Rainbow trout (*Oncorhynchus mykiss*)	150	Muscle freshness retention: 89 % (vs. 62 % control); reduced lipid oxidation.	Synthetic astaxanthin	Huang et al. (2022)
Japanese shrimp (*Penaeus japonicus*)	100	35 % inhibition of lipid peroxidation; protected phospholipid membranes.	Synthetic astaxanthin	Pan and Chien (2003)
Pacific white shrimp (*Litopenaeus vannamei*)	200	SOD activity ↑ 1.8×; CAT activity ↑ 1.5 × .	Synthetic astaxanthin	Kowshik et al. (2014)
Loach fry (*Misgurnus anguillicaudatus*)	150	Liver antioxidant enzymes ↑ 40 %; reduced oxidative damage.	Synthetic astaxanthin	Okamoto et al. (2007)
Spotted shrimp (*Penaeus* sp.)	Not specified	Survival rate ↑ 18 % under high ammonia; enhanced oxygen storage.	Synthetic astaxanthin	Samhat et al. (2023)
Giant freshwater prawn larvae (*Macrobrachium rosenbergii*)	8.25 % algae powder	Improved antioxidant capacity, lipid metabolism, and gonadal development.	Natural (algae-based)	Xu, Peng, et al. (2024)
Kuruma shrimp (*Marsupenaeus japonicus*)	100	Gill SOD activity ↑2.1×; Survival rate ↑12 %.	Synthetic astaxanthin	Pan and Chien (2003)
Black tiger shrimp (*Penaeus monodon*)	180	Hepatopancreas oxygen storage ↑31 %; higher survival under ammonia stress.	Synthetic astaxanthin	Samhat et al. (2023)

Marchetti et al. (2023) conducted single-factor experiments to extract astaxanthin from defatted liquid and fish skin. Subsequent orthogonal experiments were conducted to study its antioxidant properties and optimize the extraction process. The experiments demonstrated that astaxanthin effectively quenches singlet oxygen, inhibits free radicals, enhances stability, and prevents excessive lipid oxidation. The diphenyl bitter acyl radical is a highly stable free radical, and astaxanthin can eliminate the unpaired electron of DPPH, thereby rendering it more stable and less prone to decomposition (Guan et al., 2022). These experiments provide evidence that astaxanthin is valuable as a natural antioxidant (Table 2).

### 4.3 Astaxanthin promotes growth and antioxidant effects

The incorporation of antioxidants into feed enhances the antioxidant capacity of aquatic organisms and moderately promotes animal growth. Additionally, it aids in maintaining the stability of components within aquatic organisms and reduces nutrient loss. The commonly utilized antioxidants include vitamin A, vitamin B, vitamin C, vitamin E, glutathione, and carotenoids (Faraone et al., 2020). Samhat, Kazbar, Takache, Ismail, and Pruvost (2023) demonstrated that astaxanthin supplementation (150 mg/kg) improved oxygen storage capacity in shrimp, resulting in an 18 % increase in survival rates under high-ammonia conditions. Kowshik et al. (2014) observed that L. *vannamei* fed 200 mg/kg astaxanthin exhibited a 1.8-fold increase in superoxide dismutase (SOD) activity and a 1.5-fold increase in catalase (CAT) activity compared to controls. Sato, Sugimoto, Yamada, and Maitani (2000) reported that loach fry fed 150 mg/kg astaxanthin achieved a 25 % higher weight gain rate and a 30 % improvement in feed conversion efficiency. Okamoto et al. (2007) confirmed that astaxanthin supplementation (100 mg/kg) enhanced liver antioxidant enzyme activity in black bass by 40 %, thereby reducing oxidative damage. Moreover, Stevens, Leroux, Mowbray, and Lee (2023) investigated the antioxidant capacities of astaxanthin derived from various sources and established a correlation between the antioxidant capacity of astaxanthin and its source, structural components, and form. Notably, astaxanthin of the same type exhibits varying growth-promoting and antioxidant effects on different parts of aquatic organisms. Hafez et al. (2021) reported that astaxanthin effectively enhances the growth rate and stabilizes the survival rate of spotted shrimp. This species requires suitable temperature conditions, and the addition of astaxanthin to the feed significantly improves its survival rate. Furthermore, Xu, Zhang, et al. (2024) reported that, compared with artificial feed, supplementation with 8.25 % astaxanthin-containing algae hook shrimp powder markedly enhances the growth and gonadal development of *Litopenaeus vannamei* parent shrimp. Additionally, it promotes the antioxidant capacity and lipid metabolism of juvenile shrimp (Raihan, 2023) (Table 2).

Samhat et al. (2023) reported that astaxanthin can increase intercellular oxygen storage capacity. This effect enhances the adaptability of aquaculture animals to challenging environments such as high ammonia nitrogen and hypoxia, ultimately increasing their resistance to external stressors. Therefore, the addition of an appropriate amount of astaxanthin during the aquaculture process can effectively enhance the adaptability and stability of aquaculture animals throughout their growth, thereby promoting optimal development and growth (Table 2).

Kowshik et al. (2014) measured the effects of astaxanthin on the antioxidant, reproductive, and immune properties of L. *vannamei* by incorporating varying amounts of astaxanthin into a uniform feed. Similarly, Sato et al. (2000) divided loach fry into five groups to examine the antioxidant and growth-promoting properties of astaxanthin. These groups were administered differing levels of astaxanthin over an 8-week experiment. The results indicated that when the content of astaxanthin in the feed was maintained within the range of 100–200 mg/kg, both the volumetric weight and average weight gain rate of loaches significantly improved compared with those of the control group. Additionally, the growth-promoting efficiency and protein content of the feed were markedly greater in the experimental group. Furthermore, the activities of protease, amylase, and lipase in the intestine of loaches were measured, reaching peak values when astaxanthin was added at a dosage of 50–200 mg/kg (Chen, Huang, et al., 2023). Under the same conditions, the T-SOD activity, CAT activity, GSH content, and GSH-Px activity in the intestinal tract of loaches exceeded those of the control group. Therefore, it can be inferred that astaxanthin can effectively stimulate the growth of aquatic organisms, enhance the levels of intestinal digestive enzymes, and improve their antioxidant capacity. In a related study, Okamoto et al. (2007) conducted an experimental investigation into the effects of astaxanthin and beta-carotene on black bass. The findings of this study indicated that both astaxanthin and beta-carotene are capable of improving liver vitality and enhancing the immune system in black bass. Moreover, this study revealed that the effects of carotenoids vary across different parts of black bass. Therefore, astaxanthin is a valuable additive that efficiently enhances the growth performance of aquatic organisms whereas also improving their antioxidant capacity (Table 2).

## 5 Practical application of astaxanthin in the aquaculture industry

Astaxanthin has made significant contributions to the advancement of aquaculture, particularly in terms of pigmentation and stress resilience in aquatic animals. Furthermore, its physiological roles extend beyond simple pigmentation: dietary astaxanthin has demonstrated the ability to enhance growth performance, strengthen antioxidant defenses, improve immune responses, increase stress tolerance, elevate reproductive success, and enhance product quality across diverse aquatic taxa, including fish (e.g., *Oncorhynchus mykiss*, *Micropterus salmoides*), crustaceans (e.g., *Penaeus* spp., *E. sinensis*), and mollusks (e.g., gastropods and bivalves). Incorporating this broadened viewpoint ensures that the review precisely reflects the comprehensive role of astaxanthin in aquaculture. Recent meta-analyses (Li, Chen, et al., 2025; Li, Yu, et al., 2025) have further supported its role as a feed supplement that improves growth performance and feed conversion efficiency. Studies have demonstrated that astaxanthin not only improves the market value of aquatic animals through pigmentation but also boosts immune function and reduces oxidative stress in species such as rainbow trout and shrimp.

Astaxanthin has been shown to increase the survival rate of fish fry according to Christiansen (Ma & Zong, 2024). In their study, the elimination rate of fish fry increased when there was insufficient astaxanthin in the feed. However, when astaxanthin was adequately added to the feed, the elimination rate decreased to 10 %. These findings demonstrate the critical role of astaxanthin in the growth and development of fish fry, enhancing their ability to adapt to their environment and ultimately improving their survival rate. Therefore, astaxanthin has made significant contributions to the advancement of aquaculture.

The application of astaxanthin in the Jiangsu River Crab Pond has become a common practice aimed at enhancing the flavor and quality of commercially cultivated Chinese mitten crab (*Eriocheir sinensis*). Numerous farmers have adopted this approach based on their accumulated experiences and observations. Notably, during the shrimp farming process, when shrimp are cultivated in outer ponds, algae typically proliferate, contributing to the rich pigmentation of the shrimp's body (Zhang et al., 2020). Although shrimp acquire some astaxanthin naturally through the consumption of algae, environmental fluctuations can induce stress-related mortality in shrimp. To bolster the physical condition and stress resilience of shrimp, it is advisable to incorporate astaxanthin prior to any anticipated weather changes (Ou, Gu, Cai, & Chao, 2023), thereby facilitating the shrimp population's survival amid environmental challenges. For example, in a field trial conducted in Jiangsu Province, dietary supplementation of astaxanthin at 100 mg/kg in river crab (*Eriocheir sinensis*) resulted in a 22.7 % increase in muscle astaxanthin content, as measured by HPLC, compared to the control group. Additionally, the harvest cycle was shortened by approximately 10 days, and the average income per mu increased by 580 RMB, due to improved coloration and market price. These results highlight the tangible economic and quality benefits of applying astaxanthin in real-world aquaculture settings.

In shrimp farming, the density of shrimp within ponds is frequently elevated, whereas algae, primarily in the form of fungi, are not as plentiful. Inadequate supplementation of astaxanthin may lead to the manifestation of white and blue coloration in shrimp, which results in their shells failing to exhibit a red hue post-cooking. This negatively influences the quality of the shrimp, resulting in a diminished selling price and hindering the profitability of farmers (Chen, Huang, et al., 2023; Pan & Chien, 2003; Tran et al., 2025). It is advisable for farmers, where feasible, to administer astaxanthin 2–3 times weekly. However, for those farmers seeking to manage expenses, it is recommended to incorporate astaxanthin with other ingredients during the 30–45 days preceding the sale of shrimp. By adhering to this strategy, shrimp can maintain appropriate coloration, demonstrate robust vitality, exhibit high transportation resistance, and command higher market prices, ultimately enhancing farmers' income (Chen, Huang, et al., 2023; Pan & Chien, 2003; Samhat et al., 2023; Sun et al., 2025).

The primary market for astaxanthin currently resides in aquaculture, particularly for the cultivation of salmon and rainbow trout. Presently, 95 % of the astaxanthin available on the market is synthesized artificially. Astaxanthin, a widely distributed carotenoid, is found in various aquatic organisms, including shrimp, crabs, and birds. Although certain crustaceans possess the ability to convert other carotenoids into astaxanthin, this conversion still does not meet the organism's full requirements, necessitating its acquisition through dietary sources (Higuera-Ciapara et al., 2006; Khadilkar et al., 2023; Mutale-Joan & El Arroussi, 2023).

Artificially synthesized astaxanthin may be contaminated by other harmful substances during production and may contain unnatural isomers. Therefore, interest in the production of natural astaxanthin is increasing. Although China's aquaculture industry has experienced significant success, evidenced by consistent growth and its status as the world's largest aquaculture producer, intensification has resulted in an increase in diseases among aquatic animals. Therefore, disease prevention and healthy breeding practices have become increasingly important (Higuera-Ciapara et al., 2006; Ambati et al., 2014; Hamdi et al., 2022; Oninku et al., 2025; Li, Chen, et al., 2025; Li, Yu, et al., 2025).

The use of antibiotics for disease prevention and control is regarded as a temporary emergency measure. Broad-spectrum antibiotics possess the drawback of eliminating or inhibiting drug-sensitive bacteria whereas allowing drug-resistant pathogenic bacteria to persist. This disruption of the ecological balance within the normal microbial community in aquatic environments elevates the risk of infection among aquatic animals. Moreover, antibiotic residues in aquaculture animals pose a threat to human health. Therefore, many countries have prohibited the addition and use of antibiotics in aquaculture animal diets (European Union, 2019).

Enhancing the physical fitness of aquatic animals through immunological methods to bolster their resistance to pathogenic microorganisms presents a promising approach. Researchers globally are conducting extensive studies to identify pollution-free, residue-free, growth-promoting, and multifunctional green feed additives that can substitute for antibiotics. Investigations are underway to incorporate substances into feed that may enhance the immunity of aquatic animals. Through ongoing experimentation and exploration, various active or inactive substances have demonstrated significant biological effects. The incorporation of these substances into feed not only improves the nutritional status of aquatic animals but also markedly enhances their resistance to pathogenic microorganisms. These substances are commonly referred to as immune enhancers.

Based on comprehensive research conducted both domestically and internationally, the effects of different carotenoids on aquaculture products remain somewhat controversial. A study by Mano et al. (2018) indicated that pigments such as carotenoids exert a more pronounced coloring effect on goldfish than on shrimp.

Kohandel et al. (2022) investigated the effects of various pigments on coloration and pigment accumulation in cultured Atlantic salmon. The findings demonstrated that rainbow trout exhibit a significantly higher utilization rate of astaxanthin compared to lutein, whereas the effectiveness of astaxanthin in Atlantic salmon is less pronounced. In a related study, Jorjani, Sharif, Mirhashemi, Ako, and Tan (2019) examined the absorption and coloring effects of astaxanthin and other pigments in Atlantic salmon, concluding that the coloring effect of astaxanthin is essentially equivalent to itself. They also established a linear associated with between pigment absorption and the quantity of pigments consumed. Several reports suggest that, in comparison to astaxanthin, other pigments possess superior coloring effects on Atlantic salmon and rainbow trout. Therefore, a degree of controversy exists regarding the applicability of astaxanthin across various aquaculture species, highlighting the need for further research to assess its cost-effectiveness in different contexts.

Baker, Pfeiffer, Schöner, and Smith-Lemmon (2002) compared the coloring effects of astaxanthin from diverse sources on *Sparus aurata*. The experiment revealed no significant differences in the coloring effects of different sources or types of carotenoids on the fish. Researchers have observed that evaluating the effect of a specific pigment on the regulation of skin coloration in *S. aurata* through feeding methods alone presents challenges. However, various scholars have demonstrated that natural astaxanthin exceeds chemically synthesized astaxanthin in terms of absorption, coloring ability, and biological efficacy for aquaculture organisms. Therefore, further investigation is warranted to determine the bioavailability ratios of astaxanthin from various sources and the mechanisms of absorption and utilization of astaxanthin from different sources by aquaculture organisms (Besharat, Islami, Soltani, & Mousavi, 2024).

The feeding concentrations utilized by various researchers during astaxanthin studies differ. Nevertheless, the optimal dosage and feeding method of astaxanthin are likely to vary among different aquaculture species. To thoroughly explore the efficacy of astaxanthin application in aquaculture, it is crucial to investigate the optimal dosage and feeding method of astaxanthin. Currently, studies regarding the application of astaxanthin in aquaculture in China are limited. Only Jyonouchi, Hill, Tomita, and Good (1991) examined the effects of astaxanthin administered through red yeast on the body color and growth status of *Macrobrachium rosenbergii.*

## 6 Conclusions

Astaxanthin, a crucial dietary supplement in modern aquaculture, improves pigmentation, growth performance, antioxidant defenses, and immune responses in key aquatic species. This review examined the biological roles of astaxanthin, novel extraction methods, and its applications in improving animal health and product quality. The high cost and low output of natural astaxanthin production, along with species-specific bioavailability and manufactured isomers in commercial products, limit its utilization.

Subsequently, biotechnological breakthroughs must be utilized to create high-yield algae strains through genetic modification, optimize fermentation settings, and improve extraction procedures to increase efficiency and reduce costs. Standardizing dosage forms and delivery systems for species-specific needs improves bioavailability and effectiveness. Life-cycle assessments and economic evaluations are crucial for analyzing the sustainability and scalability of innovative production methods like recirculating aquaculture effluents.

Astaxanthin will play a more prominent role in the enhancement of sustainable aquaculture due to increased market demand and antimicrobial regulations. Research, development, and policy assistance are crucial for optimizing efficient, cost-effective, and environmentally sustainable production and consumption.

## CRediT authorship contribution statement

**Ying Zhu:** Writing – original draft, Methodology, Investigation, Formal analysis, Data curation. **Xiaoqi Li:** Writing – original draft, Visualization, Validation, Software, Investigation. **Shengjun Wu:** Writing – review & editing, Supervision, Resources, Project administration, Funding acquisition, Conceptualization.

## Declaration of competing interest

The authors declare that they have no known competing financial interests or personal relationships that could have appeared to influence the work reported in this paper.

## Data Availability

The authors do not have permission to share data.
